# Bis(pentane-2,4-dionato-κ^2^ *O*,*O*′)(1,10-phenanthroline-κ^2^ *N*,*N*′)cobalt(II)

**DOI:** 10.1107/S1600536811046216

**Published:** 2011-11-05

**Authors:** Franc Perdih

**Affiliations:** a Faculty of Chemistry and Chemical Technology, University of Ljubljana, Aškerčeva 5, PO Box 537, SI-1000 Ljubljana, Slovenia, and CO EN–FIST, Dunajska 156, SI-1000 Ljubljana, Slovenia

## Abstract

In the title compound, [Co(C_5_H_7_O_2_)_2_(C_12_H_8_N_2_)], the Co^II^ cation lies on a twofold rotation axis and is coordinated by four O atoms from two acetyl­acetonate (acac) ligands and two N atoms from a 1,10-phenanthroline (phen) ligand in a slightly distorted octa­hedral environment, with Co—O bond lengths of 2.0565 (11) and 2.0641 (11) Å and Co—N bond lengths of 2.1630 (12) Å. In the crystal, there are no significant hydrogen-bonding or π–π inter­actions.

## Related literature

For applications of metal complexes containing β-diketones, see: Garibay *et al.* (2009 ▶); Kaitner *et al.* (2008 ▶). For related cobalt(II) structures, see: Meštrović & Kaitner (2006 ▶); Riblet *et al.* (2010 ▶). For the synthetic procedure, see: Ellern & Ragsdale (1968 ▶).
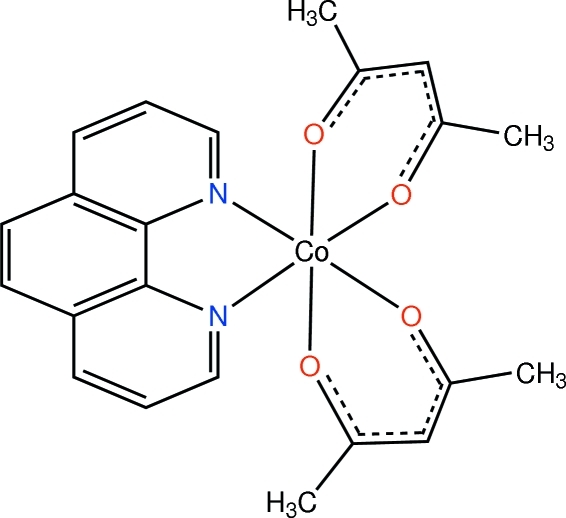


## Experimental

### 

#### Crystal data

[Co(C_5_H_7_O_2_)_2_(C_12_H_8_N_2_)]
*M* *_r_* = 437.35
Orthorhombic, 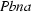

*a* = 10.2660 (2) Å
*b* = 12.6981 (3) Å
*c* = 15.5885 (3) Å
*V* = 2032.10 (7) Å^3^
*Z* = 4
Mo *K*α radiation
μ = 0.88 mm^−1^
*T* = 293 K
0.60 × 0.30 × 0.13 mm

#### Data collection

Nonius KappaCCD area-detector diffractometer
Absorption correction: multi-scan (*SCALEPACK*; Otwinowski & Minor, 1997 ▶) *T*_min_ = 0.622, *T*_max_ = 0.895
4293 measured reflections
2294 independent reflections
2022 reflections with *I* > 2σ(*I*)
*R*_int_ = 0.014

#### Refinement

*R*[*F* ^2^ > 2σ(*F* ^2^)] = 0.032
*wR*(*F* ^2^) = 0.089
*S* = 1.08
2294 reflections
134 parameters
H-atom parameters constrained
Δρ_max_ = 0.28 e Å^−3^
Δρ_min_ = −0.36 e Å^−3^

### 

Data collection: *COLLECT* (Hooft, 1998 ▶); cell refinement: *DENZO* (Otwinowski & Minor, 1997 ▶); data reduction: *SCALEPACK* (Otwinowski & Minor, 1997 ▶); program(s) used to solve structure: *SHELXS97* (Sheldrick, 2008 ▶); program(s) used to refine structure: *SHELXL97* (Sheldrick, 2008 ▶); molecular graphics: *ORTEP-3* (Farrugia, 1997 ▶); software used to prepare material for publication: *WinGX* (Farrugia, 1999 ▶) and *publCIF* (Westrip, 2010 ▶).

## Supplementary Material

Crystal structure: contains datablock(s) I, global. DOI: 10.1107/S1600536811046216/pv2467sup1.cif

Structure factors: contains datablock(s) I. DOI: 10.1107/S1600536811046216/pv2467Isup2.hkl

Additional supplementary materials:  crystallographic information; 3D view; checkCIF report

## Figures and Tables

**Fig. 1.**
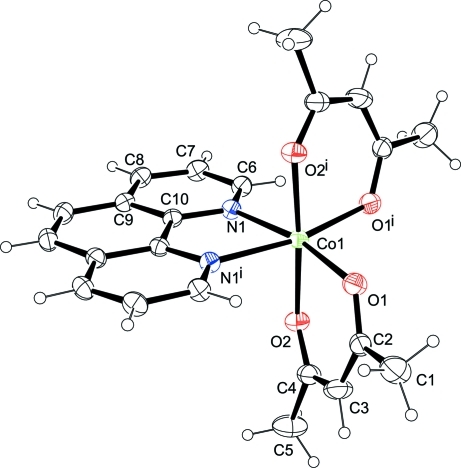
The molecular structure of the title complex showing the numbering scheme and displacement ellipsoids drawn at the 30% probability level. Symmetry code: i = x, -y+1/2, -z+1.

## supplementary crystallographic information

### Comment

Metal β-diketonate compounds attracted great interest, because metal complexes of β-diketonate derivatives are useful building blocks for design of porous and supramolecular materials and can be good precursors in metal-organic chemical vapour deposition (MOCVD) (Garibay *et al.*, 2009; Kaitner *et al.* 2008).

In the title molecule (Fig. 1), the cobalt(II) cation lies on a twofold axis, and is surrounded by six donor atoms arranged at the vertices of a distorted octahedron. The coordination polyhedron of Co^II^ is formed by four oxygen atoms of two symmetry related 2,4-pentanedionato ligands and two nitrogen atoms of 1,10-phenantroline. Consequently, the octahedron is defined by two symmetry-independent cobalt-oxygen bonds Co–O1 2.0565 (11) Å (in *trans* position to N1 of the phen ligand) and Co–O2 2.0641 (11) Å (in *cis* position to N1 of the phen ligand) and a cobalt-nitrogen bond Co–N1 2.1630 (12) Å. These bond lengths are similar with the ones observed in adducts (1,10-phenanthroline)bis(1,3-diphenyl-1,3-propanedionato)cobalt(II) (Meštrović & Kaitner 2006) and (2,2'-bipyridine)bis(4,4,4-trifluoro-1-(2-thienyl)-1,3-butanedionato)cobalt(II) (Riblet *et al.*, 2010). The discrepancy of the coordination polyhedron of Co^II^ from the ideal octahedral arrangement is well illustrated by the angles O1–Co–O2 and N1–Co–N1 of 87.62 (4)° and 77.20 (6)°, respectively. In the crystal structure, there are no hydrogen-bonding or π–π interactions.

### Experimental

(1,10-Phenanthroline-κ^2^*N*,*N*')bis(2,4-pentanedionato-κ^2^*O*,*O*')cobalt(II) was prepared according to the published procedure (Ellern & Ragsdale, 1968). 0.25 mmol (0.064 mg) of bis(2,4-pentanedionato-κ^2^ O,*O*')cobalt(II) was disolved in 5 ml of warm chloroform and than 0.25 mmol (0.045 mg) of 1,10-phenantroline was added. Orange crystals suitable for single-crystal *X*–ray diffraction were obtained after slow evaporation.

### Refinement

Although H atoms were visible in a difference Fourier map they were treated in riding mode in geometrically idealized positions, with C—H = 0.93 (aromatic and alkenyl) or 0.96 Å (CH_3_), and with *U*_iso_(H) = *kU*_eq_(C), where *k* = 1.5 for methyl groups, which were permitted to rotate but not to tilt, and 1.2 for all other H atoms. To improve the refinement results, one reflection with too high value of *δ*(*F*^2^)/e.s.d. and with *F*_o_^2^ < *F*_c_^2^ was excluded from the refinement.

### Crystal data

**Table:**

[Co(C_5_H_7_O_2_)_2_(C_12_H_8_N_2_)]	*F*(000) = 908
*M**_r_* = 437.35	*D*_x_ = 1.43 Mg m^−^^3^
Orthorhombic, *P**b**n**a*	Mo *K*α radiation, λ = 0.71073 Å
Hall symbol: -P 2ac 2b	Cell parameters from 2617 reflections
*a* = 10.2660 (2) Å	θ = 0.4–27.5°
*b* = 12.6981 (3) Å	µ = 0.88 mm^−^^1^
*c* = 15.5885 (3) Å	*T* = 293 K
*V* = 2032.10 (7) Å^3^	Block, orange
*Z* = 4	0.6 × 0.3 × 0.13 mm

### Data collection

**Table:**

Nonius KappaCCD area-detector diffractometer	2294 independent reflections
graphite	2022 reflections with *I* > 2σ(*I*)
Detector resolution: 0.055 pixels mm^-1^	*R*_int_ = 0.014
φ and ω scans	θ_max_ = 27.5°, θ_min_ = 3.5°
Absorption correction: multi-scan (*SCALEPACK*; Otwinowski & Minor, 1997)	*h* = −13→13
*T*_min_ = 0.622, *T*_max_ = 0.895	*k* = −16→16
4293 measured reflections	*l* = −20→20

### Refinement

**Table:**

Refinement on *F*^2^	Primary atom site location: structure-invariant direct methods
Least-squares matrix: full	Secondary atom site location: difference Fourier map
*R*[*F*^2^ > 2σ(*F*^2^)] = 0.032	Hydrogen site location: inferred from neighbouring sites
*wR*(*F*^2^) = 0.089	H-atom parameters constrained
*S* = 1.08	*w* = 1/[σ^2^(*F*_o_^2^) + (0.0472*P*)^2^ + 0.6276*P*] where *P* = (*F*_o_^2^ + 2*F*_c_^2^)/3
2294 reflections	(Δ/σ)_max_ < 0.001
134 parameters	Δρ_max_ = 0.28 e Å^−^^3^
0 restraints	Δρ_min_ = −0.36 e Å^−^^3^

### Special details

**Table:**

Experimental. 346 frames in 4 sets of φ scans + ω scans. Rotation/frame = 2.0 °. Crystal-detector distance = 34.7 mm. Measuring time = 20 s/°.
Geometry. All s.u.'s (except the s.u. in the dihedral angle between two l.s. planes) are estimated using the full covariance matrix. The cell s.u.'s are taken into account individually in the estimation of s.u.'s in distances, angles and torsion angles; correlations between s.u.'s in cell parameters are only used when they are defined by crystal symmetry. An approximate (isotropic) treatment of cell s.u.'s is used for estimating s.u.'s involving l.s. planes.
Refinement. Refinement of *F*^2^ against ALL reflections. The weighted *R*-factor *wR* and goodness of fit *S* are based on *F*^2^, conventional *R*-factors *R* are based on *F*, with *F* set to zero for negative *F*^2^. The threshold expression of *F*^2^ > 2σ(*F*^2^) is used only for calculating *R*-factors(gt) *etc*. and is not relevant to the choice of reflections for refinement. *R*-factors based on *F*^2^ are statistically about twice as large as those based on *F*, and *R*- factors based on ALL data will be even larger.

### Fractional atomic coordinates and isotropic or equivalent isotropic displacement parameters (Å^2^)

**Table:**

	*x*	*y*	*z*	*U*_iso_*/*U*_eq_	
Co1	−0.01460 (3)	0.25	0.5	0.03014 (12)	
N1	0.15007 (12)	0.34681 (9)	0.46431 (8)	0.0314 (3)	
O1	−0.14810 (11)	0.14898 (9)	0.55387 (7)	0.0427 (3)	
O2	−0.00799 (11)	0.32708 (9)	0.61650 (7)	0.0401 (3)	
C1	−0.2481 (2)	0.03967 (18)	0.65656 (14)	0.0662 (6)	
H1A	−0.3184	0.0319	0.6165	0.099*	
H1B	−0.2827	0.0488	0.7133	0.099*	
H1C	−0.1943	−0.0222	0.6551	0.099*	
C2	−0.16730 (15)	0.13500 (14)	0.63257 (10)	0.0417 (4)	
C3	−0.1192 (2)	0.19883 (16)	0.69833 (11)	0.0536 (5)	
H3	−0.1388	0.1791	0.7543	0.064*	
C4	−0.04462 (18)	0.28934 (14)	0.68732 (10)	0.0423 (4)	
C5	−0.0002 (2)	0.34879 (19)	0.76625 (12)	0.0711 (7)	
H5A	0.0868	0.3276	0.7808	0.107*	
H5B	−0.0576	0.3334	0.8132	0.107*	
H5C	−0.0016	0.4231	0.7547	0.107*	
C6	0.14845 (15)	0.44271 (11)	0.43038 (10)	0.0360 (3)	
H6	0.0684	0.4745	0.4198	0.043*	
C7	0.26196 (16)	0.49794 (11)	0.40985 (11)	0.0402 (4)	
H7	0.257	0.5652	0.3864	0.048*	
C8	0.38011 (15)	0.45233 (12)	0.42452 (10)	0.0383 (3)	
H8	0.4563	0.4877	0.4099	0.046*	
C9	0.38654 (14)	0.35112 (11)	0.46198 (9)	0.0329 (3)	
C10	0.26736 (14)	0.30172 (10)	0.48049 (9)	0.0295 (3)	
C11	0.50593 (15)	0.29880 (15)	0.48177 (11)	0.0384 (4)	
H11	0.5847	0.3317	0.4696	0.046*	

### Atomic displacement parameters (Å^2^)

**Table:**

	*U*^11^	*U*^22^	*U*^33^	*U*^12^	*U*^13^	*U*^23^
Co1	0.03095 (18)	0.03099 (18)	0.02847 (17)	0	0	0.00224 (10)
N1	0.0328 (6)	0.0283 (6)	0.0331 (6)	0.0006 (5)	0.0004 (5)	0.0019 (5)
O1	0.0419 (6)	0.0490 (7)	0.0372 (6)	−0.0132 (5)	−0.0024 (5)	0.0051 (5)
O2	0.0501 (6)	0.0370 (6)	0.0331 (5)	−0.0006 (4)	0.0016 (5)	−0.0016 (4)
C1	0.0646 (12)	0.0776 (14)	0.0563 (12)	−0.0301 (11)	−0.0085 (9)	0.0266 (11)
C2	0.0338 (7)	0.0511 (9)	0.0401 (8)	−0.0038 (7)	−0.0014 (6)	0.0124 (7)
C3	0.0636 (12)	0.0658 (12)	0.0315 (8)	−0.0112 (9)	0.0035 (8)	0.0083 (8)
C4	0.0484 (9)	0.0463 (9)	0.0320 (8)	0.0059 (8)	−0.0009 (7)	−0.0019 (7)
C5	0.1033 (19)	0.0719 (15)	0.0381 (10)	−0.0125 (12)	−0.0019 (10)	−0.0115 (10)
C6	0.0378 (7)	0.0312 (7)	0.0389 (8)	0.0028 (6)	0.0019 (6)	0.0039 (6)
C7	0.0483 (9)	0.0304 (7)	0.0420 (9)	−0.0016 (6)	0.0046 (7)	0.0064 (6)
C8	0.0391 (8)	0.0354 (8)	0.0404 (8)	−0.0062 (6)	0.0074 (6)	0.0032 (6)
C9	0.0344 (7)	0.0332 (7)	0.0311 (7)	−0.0022 (6)	0.0030 (6)	−0.0014 (6)
C10	0.0318 (7)	0.0283 (7)	0.0285 (6)	−0.0001 (5)	0.0009 (5)	−0.0011 (5)
C11	0.0315 (7)	0.0413 (10)	0.0424 (8)	−0.0040 (6)	0.0037 (6)	−0.0001 (7)

### Geometric parameters (Å, °)

**Table:**

Co1—O1	2.0565 (11)	C3—H3	0.93
Co1—O1^i^	2.0565 (11)	C4—C5	1.514 (2)
Co1—O2	2.0641 (11)	C5—H5A	0.96
Co1—O2^i^	2.0641 (11)	C5—H5B	0.96
Co1—N1^i^	2.1630 (12)	C5—H5C	0.96
Co1—N1	2.1630 (12)	C6—C7	1.397 (2)
N1—C6	1.3277 (18)	C6—H6	0.93
N1—C10	1.3570 (18)	C7—C8	1.363 (2)
O1—C2	1.2551 (19)	C7—H7	0.93
O2—C4	1.261 (2)	C8—C9	1.413 (2)
C1—C2	1.514 (2)	C8—H8	0.93
C1—H1A	0.96	C9—C10	1.4049 (19)
C1—H1B	0.96	C9—C11	1.428 (2)
C1—H1C	0.96	C10—C10^i^	1.448 (3)
C2—C3	1.397 (3)	C11—C11^i^	1.363 (4)
C3—C4	1.392 (3)	C11—H11	0.93
			
O1—Co1—O1^i^	96.41 (7)	C4—C3—H3	117.2
O1—Co1—O2	87.62 (4)	C2—C3—H3	117.2
O1^i^—Co1—O2	94.90 (4)	O2—C4—C3	125.87 (15)
O1—Co1—O2^i^	94.90 (4)	O2—C4—C5	115.61 (17)
O1^i^—Co1—O2^i^	87.62 (4)	C3—C4—C5	118.51 (16)
O2—Co1—O2^i^	176.23 (6)	C4—C5—H5A	109.5
O1—Co1—N1^i^	93.51 (5)	C4—C5—H5B	109.5
O1^i^—Co1—N1^i^	168.64 (4)	H5A—C5—H5B	109.5
O2—Co1—N1^i^	91.00 (4)	C4—C5—H5C	109.5
O2^i^—Co1—N1^i^	86.05 (4)	H5A—C5—H5C	109.5
O1—Co1—N1	168.64 (4)	H5B—C5—H5C	109.5
O1^i^—Co1—N1	93.51 (5)	N1—C6—C7	122.76 (14)
O2—Co1—N1	86.05 (4)	N1—C6—H6	118.6
O2^i^—Co1—N1	91.00 (4)	C7—C6—H6	118.6
N1^i^—Co1—N1	77.20 (6)	C8—C7—C6	119.36 (13)
C6—N1—C10	118.17 (12)	C8—C7—H7	120.3
C6—N1—Co1	127.88 (10)	C6—C7—H7	120.3
C10—N1—Co1	113.95 (9)	C7—C8—C9	119.81 (14)
C2—O1—Co1	126.30 (10)	C7—C8—H8	120.1
C4—O2—Co1	125.47 (11)	C9—C8—H8	120.1
C2—C1—H1A	109.5	C10—C9—C8	116.76 (13)
C2—C1—H1B	109.5	C10—C9—C11	119.70 (13)
H1A—C1—H1B	109.5	C8—C9—C11	123.54 (14)
C2—C1—H1C	109.5	N1—C10—C9	123.11 (12)
H1A—C1—H1C	109.5	N1—C10—C10^i^	117.45 (7)
H1B—C1—H1C	109.5	C9—C10—C10^i^	119.44 (8)
O1—C2—C3	125.43 (15)	C11^i^—C11—C9	120.87 (9)
O1—C2—C1	116.13 (16)	C11^i^—C11—H11	119.6
C3—C2—C1	118.43 (16)	C9—C11—H11	119.6
C4—C3—C2	125.66 (15)		
			
O1—Co1—N1—C6	−143.2 (2)	C1—C2—C3—C4	−179.5 (2)
O1^i^—Co1—N1—C6	7.72 (13)	Co1—O2—C4—C3	−13.7 (3)
O2—Co1—N1—C6	−86.96 (13)	Co1—O2—C4—C5	165.38 (13)
O2^i^—Co1—N1—C6	95.39 (13)	C2—C3—C4—O2	−0.7 (3)
N1^i^—Co1—N1—C6	−178.89 (15)	C2—C3—C4—C5	−179.73 (19)
O1—Co1—N1—C10	36.0 (3)	C10—N1—C6—C7	0.8 (2)
O1^i^—Co1—N1—C10	−173.10 (10)	Co1—N1—C6—C7	180.00 (11)
O2—Co1—N1—C10	92.22 (10)	N1—C6—C7—C8	0.3 (2)
O2^i^—Co1—N1—C10	−85.43 (10)	C6—C7—C8—C9	−1.4 (2)
N1^i^—Co1—N1—C10	0.29 (7)	C7—C8—C9—C10	1.4 (2)
O1^i^—Co1—O1—C2	−113.29 (15)	C7—C8—C9—C11	−178.19 (15)
O2—Co1—O1—C2	−18.62 (14)	C6—N1—C10—C9	−0.9 (2)
O2^i^—Co1—O1—C2	158.57 (14)	Co1—N1—C10—C9	179.85 (11)
N1^i^—Co1—O1—C2	72.24 (14)	C6—N1—C10—C10^i^	178.45 (15)
N1—Co1—O1—C2	37.5 (3)	Co1—N1—C10—C10^i^	−0.82 (19)
O1—Co1—O2—C4	19.03 (14)	C8—C9—C10—N1	−0.2 (2)
O1^i^—Co1—O2—C4	115.26 (13)	C11—C9—C10—N1	179.37 (14)
N1^i^—Co1—O2—C4	−74.45 (13)	C8—C9—C10—C10^i^	−179.54 (15)
N1—Co1—O2—C4	−151.54 (14)	C11—C9—C10—C10^i^	0.1 (2)
Co1—O1—C2—C3	12.6 (3)	C10—C9—C11—C11^i^	0.2 (3)
Co1—O1—C2—C1	−166.56 (13)	C8—C9—C11—C11^i^	179.75 (19)
O1—C2—C3—C4	1.4 (3)		

Symmetry codes: (i) *x*, −*y*+1/2, −*z*+1.
